# APPRECIATIVE INQUIRY WORKSHOP TO DEVELOP A DYADIC INTERVENTION FOR PARENTS AND ADULT CHILDREN COPING WITH DEMENTIA

**DOI:** 10.1093/geroni/igae098.1864

**Published:** 2024-12-31

**Authors:** Joan Monin, Heidi Gil, Jenna Weiss, Ellen Sue Moses, Lucy Rosenblatt

**Affiliations:** Yale School of Public Health, New Haven, Connecticut, United States; LiveWell Institute, Southington, Connecticut, United States; LiveWell Institute, Southington, Connecticut, United States; LiveWell Institute, Southington, Connecticut, United States; LiveWell Institute, Southington, Connecticut, United States

## Abstract

Including the voices of people with lived experience should be a requisite step in designing dyadic intervention studies to ensure that study design and measures are acceptable, feasible, and suitable. I will discuss my experience as a researcher participating in a LiveWell Institute Appreciative Inquiry Workshop with the Empowering Partnership Network (EPN), a diverse group of partners that connect and influence dementia research and innovation, advocacy and awareness, and the design and development of systems and spaces intended for people living with dementia. The EPN consists of people living with dementia, care partners, and other partners (researchers, policy makers, practitioners). The goal of the workshop was to begin to develop an attachment-based communication intervention for adult children and their parents coping with dementia. First, we took time to meet each other and identify each other’s strengths. Second, we set guiding principles to help each other be at our best (e.g. not speaking on anyone’s behalf; giving enough time to speak). Third, in the discovery phase, I shared why this work is important and what we know about the science of Attachment theory, dementia, and parent-child relationships. I described my Stage 0 findings and provided examples of other evidenced-based interventions on which we could build. Fourth, in the dream phase, the group shared their hopes about the ideal intervention. Fifth, in the design phase, the group suggested ways of recruiting, what the materials were and what the experience should be. Finally, in the destiny phase, we established the next action steps.

